# Molecular Imaging of Dopamine Partial Agonists in Humans: Implications for Clinical Practice

**DOI:** 10.3389/fpsyt.2022.832209

**Published:** 2022-04-06

**Authors:** Xenia M. Hart, Christian N. Schmitz, Gerhard Gründer

**Affiliations:** ^1^Department of Molecular Neuroimaging, Central Institute of Mental Health, Medical Faculty Mannheim, University of Heidelberg, Mannheim, Germany; ^2^Department of Psychiatry and Psychotherapy, Central Institute of Mental Health, Medical Faculty Mannheim, University of Heidelberg, Mannheim, Germany

**Keywords:** dopamine partial agonists, brexpiprazole, cariprazine, aripiprazole, positron emission tomography, molecular neuroimaging

## Abstract

Positron emission tomography (PET) has been used since the late 1980s for the assessment of relationships between occupancy of D_2/3_ receptors by antipsychotic drugs in the human brain and the clinical effects and side effects of these compounds in patients. It is now well established for most D_2/3_ antagonists, both of the first and the second generation, that the ideal occupancy of their target receptors is between approximately 65 and 80%. If the occupancy is below 65%, the probability of treatment response is reduced, if the occupancy is higher than 80%, the risk for extrapyramidal side-effects increases substantially. However, partial agonist antipsychotics behave different from these rules. It has been shown for all three available drugs of this class (aripiprazole, brexpiprazole, cariprazine) that, due to their special pharmacology, a very high target engagement (>90%) not only is not harmful but represents a prerequisite for antipsychotic efficacy. The available PET studies for these drugs are reviewed in this work. It is demonstrated that optimal plasma levels for partial agonist antipsychotics can be derived from these studies, which can guide individual treatment in routine patient care.

## Introduction

Determination of clinically useful and rational doses of antipsychotics represents the application of neuroimaging that has had the largest impact on clinical practice in psychiatry (1–3). Molecular imaging with positron emission tomography (PET) is now a routine tool for development of new compounds of this class (3). All antipsychotic agents that are currently in use for the treatment of psychotic disorders, such as schizophrenia, are either antagonists or partial agonists at dopamine D_2/3_ receptors. Assessment of occupancy (target engagement, TE) of these receptors by antipsychotics helped in establishing relationships between TE and antipsychotic doses and their respective plasma concentrations. Studies of the clinical effects and side effects as a function of TE facilitated not only the understanding of antipsychotic drug action, but also the rational dosing of these compounds, which can be further improved when dosing is guided by Therapeutic Drug Monitoring [TDM; (2)]. Assessment of TE with PET or single photon emission computed tomography (SPECT) is based on the concept that the experimental pharmaceutical displaces the radioligand, which binds to the target at trace concentrations. The extent of this displacement is related to the baseline binding of the radioligand in its unblocked state. Because it is often not feasible to study patients with schizophrenia in medication-free state, patients are usually studied in blocked state only (which means that they are treated with the experimental drug). Unblocked baseline data are taken from healthy volunteers, assuming that patients in the untreated state and controls differ only marginally in receptor availability. The radioactivity in the region of interest in the blocked vs. the unblocked state then. provides the target occupancy (in%) as follows (2):


(1) Occupancy[%]=100-[(Tracerbinding/blockedTracerBinding)unblocked×100]

**FIGURE 1:**
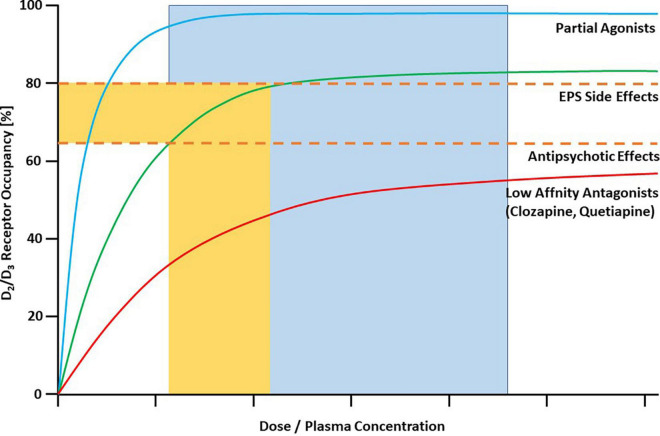
Characteristic binding curves of antipsychotic drugs in human striatum as measured with PET. Dashed lines represent threshold occupancy values for EPS (80%) and antipsychotic effects (65%). Most antipsychotics, including most of the SGAs, are characterized by the green line. They reach optimal occupancy (65–80%) in a “therapeutic window” of corresponding plasma concentrations. Antipsychotics with low affinity for D_2_/D_3_ receptors are described by the red line. Even at very high plasma concentrations they usually do not cross the 80% threshold for EPS. They exert antipsychotic effects despite relatively low occupancy in the striatum. All clinically available partial agonist antipsychotics are characterized by the blue binding curve. They have antipsychotic effects only at almost total saturation of D_2_/D_3_ receptors (in the flat part of the curve), represented by the blue area. The upper threshold is not sharply defined. Copyright © 1969, Elsevier. From (1).

Farde et al. in their pioneering early PET studies from the late 1980s demonstrated that clinically effective doses of first-generation antipsychotics (e.g., haloperidol) occupy D_2/3_ dopamine receptors in the striatum of patients with schizophrenia in the range between 65 and 90% (4). These authors also suggested a “therapeutic window” between 65 and 80% striatal dopamine D_2/3_ receptor occupancy for antipsychotic drug action, implying a “ceiling” of about 65% occupancy for sufficient treatment response, although such a high occupancy does not necessarily mean that every patient sufficiently improves. The risk for extrapyramidal side-effects (EPS) increases above a striatal D_2/3_ receptor occupancy of 80%. These relationships also apply to most of the second-generation antipsychotics (5). However, there are certain exceptions to those general rules (6). Antipsychotics with low affinity for D_2_-like dopamine receptors such as clozapine and quetiapine even at very high doses or plasma concentrations practically never occupy striatal D_2/3_ receptors to an extent that is associated with EPS (7, 8). Partial agonists at D_2/3_ receptors, on the other hand, have a completely different binding pattern at their main targets. At clinically effective doses, they almost completely occupy D_2/3_ receptors, an observation that has been made first for aripiprazole (9). This unique feature is explained by the pharmacological properties of partial agonists with low intrinsic activity (10). Figure 1 depicts the different prototypic patterns of target engagement of the available antipsychotic drugs at striatal D_2/3_ dopamine receptors as a function of their plasma concentrations.

Here, we summarize the literature on molecular imaging studies with the available partial agonists, aripiprazole, brexpiprazole, and cariprazine. We show that these studies, especially when target engagement is related to plasma concentrations of the respective drug, can guide rational dosing and Therapeutic Drug Monitoring of these compounds.

Aripiprazole was the first D_2/3_ dopamine partial agonist that was approved for the treatment of schizophrenia (United States: 2002). It was later approved for various other indications including mania and major depression (adjunctive treatment). Aripiprazole binds with very high affinity (in the low nanomolar range) to D_2_ and somewhat lesser affinity to D_3_ receptors. At both receptors it acts as a partial agonist with low intrinsic activity. Aripiprazole is also a partial agonist at the 5-HT_1A_ and an antagonist at the 5-HT_2A_ serotonin receptor. It has an elimination half-life of 60–80 h. Its main active metabolite, dehydroaripiprazole, has a similar receptor binding profile, and it amounts to up to 40% of the parent concentrations (11).

Brexpiprazole is approved for the treatment of schizophrenia (United States: 2015) and as an adjunctive treatment for major depression. It has a binding profile very similar to the one of its predecessor aripiprazole, with somewhat lower intrinsic activity at D_2_ and D_3_ receptors. Brexpiprazole has an elimination half-life of approximately 90 h. Its main metabolite (DM-3411) amounts to 23–48% of the parent compound, but it does not contribute to the pharmacodynamic effects, because it does not pass the blood-brain barrier (12).

Cariprazine received FDA approval for the treatment of schizophrenia in 2015. It has partial agonist activity at dopamine D_2/3_ receptors, with and six- to eightfold higher affinity for human dopamine D_3_ over D_2_ receptors. Like aripiprazole and brexpiprazole, cariprazine is a partial agonist at the 5-HT_1A_ and an antagonist at the 5-HT_2A_ serotonin receptor. The elimination half-life of the parent compound is 50–120 h. However, cariprazine has two active metabolites, N-desmethyl cariprazine (DCAR) and NN-didesmethyl cariprazine (DDCAR). DDCAR is eliminated with a half-life of 2–3 weeks. At steady-state, it significantly contributes to the antipsychotic activity of the drug (13, 14).

## Methods

### Search Strategy

In September 2021 (last updated 08.12.2021), four electronic databases (PsycINFO, Medline via PubMed, Cochrane CENTRAL, Web of Science) were systematically searched for relevant articles without restrictions in language or publication date. Keywords included the respective psychotropic drug (aripiprazole, brexpiprazole or cariprazine) and PET/SPECT. Studies in humans and non-human primates were included. Only full-text articles were taken into consideration, abstracts were excluded.

### Calculation of EC_90_ Values

The available literature was screened for papers that reported D_2/3_ dopamine receptor occupancy values of the respective drug in relation to administered doses. Both studies in healthy volunteers and in patients were acceptable. Special emphasis was put on studies that also reported plasma or serum drug concentrations, because they usually allow the calculation of an “effective concentration 50” (EC_50_), which is the concentration predicted to provide 50% of the maximum attainable receptor occupancy. This is a constant characterizing an individual drug. It is related to the maximum attainable receptor occupancy (E_*max*_) and the plasma concentration of the drug (C) that is associated with a measured receptor occupancy according to the law of mass action (Michaelis-Menten kinetics):


(2) Occupancy[%]=(E×max[C])/(EC+50[C])

From the experimentally determined EC_50_ values, an EC_90_ value can be calculated according to the following equations (maximum attainable receptor occupancy is less than 100%; unconstrained model):


(3) 90×(EC+50[C])=E×max[C]

(4) 90×EC+5090[C]=E×max[C]

(5) 90×EC=50E×max[C]-90[C]

Assuming that the maximum attainable receptor occupancy is 100% (i.e., all available receptors can be occupied by the drug; constrained model), EC_90_ is then:


(6) EC=90(90×EC)50/10

Uchida et al. (15) demonstrated that the relationship between D_2/3_ dopamine receptor occupancy and the respective plasma levels are in some cases better described by an unconstrained model. The constrained model assumes that all dopamine D_2/3_ receptors (100%) can be occupied by the antipsychotic. For most antipsychotics, E_*max*_ values derived with an unconstrained model are close to 100%, and therefore EC_50_ values estimated from the constrained and the unconstrained model do not substantially differ. For example, for haloperidol the EC_50_ estimated from the unconstrained model was 0.32 and 0.70 ng/ml, when E_*max*_ was constrained to 100% (15). For olanzapine, the respective values are 7 and 10 ng/ml, and for risperidone 5 and 8 ng/ml. For compounds with a low affinity to D_2/3_ receptors such as clozapine, the situation is more complicated. Here, the experimentally determined E_*max*_ values are far below 100%. Using an unconstrained model, Uchida et al. (15) calculated a maximum attainable receptor occupancy for clozapine of only 60%, with a respective EC_50_ of 105 ng/ml. The constrained model provided an EC_50_ value of 483 ng/ml. Biologically, it makes no sense to believe that clozapine does not occupy more than 60% of striatal D_2/3_ dopamine receptors. In monkeys, high doses of clozapine occupy more than 80% of D_2/3_ receptors (16). Almost all PET studies that determined D_2/3_ dopamine receptor occupancy by clozapine used [^11^C]raclopride as the radiotracer (15). In our own study with [^18^F]fallypride as the radiotracer, we calculated, using an unconstrained model, an E_*max*_ close to complete receptor saturation, and respective EC_50_ values of 950 ng/mL for the putamen and 582 ng/ml for the caudate (7). These values seem to be biologically and especially clinically more meaningful, since the therapeutic reference range for clozapine is 350 – 600 ng/ml (17), and even much higher plasma concentrations are tolerated without extrapyramidal side-effects (7).

For the purpose of this paper, it seems feasible to work with EC_90_ values that are derived from a constrained model. All available D_2/3_ partial agonist antipsychotics are high affinity compounds that occupy their main molecular target close to saturation at doses used in clinical practice. Differences in EC_90_ values calculated from constrained versus unconstrained models might therefore be negligible. It is proposed here that the EC_90_ values determined experimentally with molecular (in almost all cases PET) imaging represent the lower threshold of a therapeutic reference range to be used for TDM.

## Molecular Imaging of Dopamine Partial Agonists

### Aripiprazole

**FIGURE 2:**
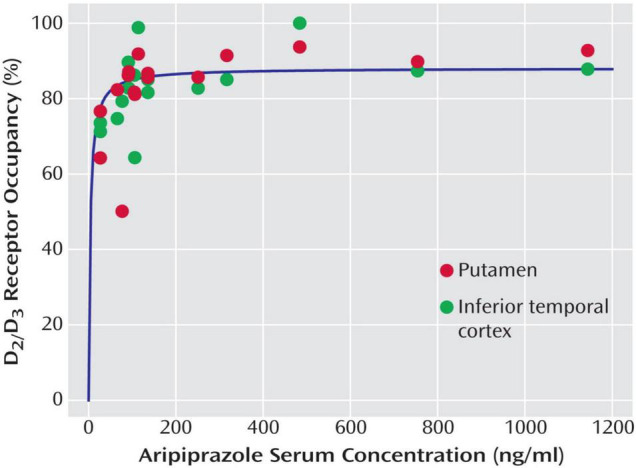
Relationship between aripiprazole serum levels and dopamine D2/D3 receptor occupancy in the putamen and the inferior temporal cortex (representative of cortical binding due to high D2/D3 receptor density) in 16 patients with schizophrenia and schizoaffective disorder receiving therapeutic doses of aripiprazole. Copyright © American Psychiatric Association. From (18).

For aripiprazole, nine PET studies in human subjects are available that report D_2/3_ receptor occupancy values (9, 18–26) (Table 1). However, only two of them report ED_50_ values [or individual plasma concentrations, from which an ED_50_ value was derived: (18, 26); Figure 2].

**TABLE 1:** PET studies reporting D_2_ receptor occupancy and aripiprazole (ARI) blood concentrations.

No	Author, year	PET tracer	Design	Subjects	*Mean Dose (range) [mg/day]*	Mean ARI Conc. (range) [ng/ml]	Mean Receptor occupancy (%)	EC_50_ [ng/ml]	EC_90_ (estimated from EC_50_) [ng/ml]	Comment
1	(9)	[^11^C]raclopride	Cohort study, dose response PET scans of fixed doses of ARI taken for 14 days, trough samples analyzed by HPLC with UV detection	*N* = 15; healthy volunteers; age 32 ± 9; 100% males	10 ± 12.8 (0.5–30)	NA (only in diagram)	D_2/3_: 66.8 ± 25.0 (c); 66.9 ± 21.59 (p)	NA	NA	Hyperbolic relation between peak ARI conc. and D_2_ occup. (p)
2	(23); (24) (same cohort)	[^11^C]raclopride, [^18^F]setoperone, [^11^C]WAY100635	RCT, 3 PET scans after ARI taken for 14 days; diagnosis acc. to DSM-4. Peak levels measured with LC/MS, clinical efficacy assessments	*N* = 12; SCZ or SD; age 31 ± 7; 75% males	18.8 ± 7.7 (10–30)	220.8 ± 179.0	D_2/3_: 86.6 ± 3.7 (p), 92.9 ± 5.7 (c), 91.0 ± 4.0 (cs); 5-HT_2_: 54.0 ± 15.3 (tc), 59.4 ± 12.9 (fc); 5-HT_1A_: 16.2 ± 14.3 (tc), 16.5 ± 13.8 (fc)	NA	NA	ARI and DARI conc. correlated with D_2_ occup. (p and s). No corr. between occup. and clinical or well-being scores. EPS in 2 patients with occup. >90%
3	(18)	[^18^F]fallypride	Cohort study with unmedicated vs. medicated patients, trough serum concentrations in steady-state measured with HPLC	*N* = 16/8 (medicated/unmedicated); SCZ or SD (DSM-4); age 30; 94% males	18.8 ± 7.2 (5–30)	245 ± 307	D_2/3_: 83 ± 1 (p), 84 ± 1 (c), 85 ± 7 (t)	10 ± 4 (p) 9 ± 4 (c)	90 (p), 81 (c)	Complete occup. with ARI conc. >100–150 ng/ml. Lower EC_50_ in thalamus (6 ± 2 ng/mL)
4	(20)	[^18^F]fallypride	Cohort study, fixed doses of ARI taken for min. 14 days, serum conc. measured with RP LC with UV, clinical efficacy assessments	*N* = 19; SCZ or SD (DSM-4); age 29; 79% males	13.9 ± 11 (2–40)	NA (excl. in analysis)	D_2/3_: NA 79.8 ± 14.8 (s) in 15 mg	ED_80_ 5.63 ± 1.0 (s) approx. 100 ng/ml	NA	Dose correlated with ARI conc., PANSS positive scale corr. with D_2_ occup. (s). No EPS.
5	(19)	[^11^C]raclopride, L-[ß-^11^C]DOPA	Cohort study on dopamine synthesis capacity, PET scans after single dose of ARI, serum conc. measured with LC/MS	*N* = 12; healthy volunteers; age 24.1 ± 3.2; 100% males	5.3 ± 2.3 (3–9)	23.8 ± 11.3	D_2/3_: 67.2 ± 9.7 (c), 64.3 ± 8.9 (p)	NA	NA	No changes in dopamine synthesis capacity.
6	(21)	[^11^C]raclopride	RCT, single dose of aripiprazole after fasting, sampling up to 120 h	*N* = 18; healthy volunteers; age 22.9 ± 2.4; 100% males	12.7 ± 11.5 (2–30)	Peak: 3.4 ± 0.9 per mg	D_2/3_: 61.7 ± 21.2 (s)	11.1 (s)	99.9 (s)	Values reported for PK model; PK/PD model estimates EC_90_ of 77.4 ng/mL (s)
7	(26)	[^11^C]raclopride, [^11^C]FLB457	Cohort single dose study on extrastriatal binding of ARI, peak conc. measured with LC/MS	*N* = 11; healthy volunteers; age 23.7 ± 4.0; 100% males	6	29.4 ± 4.8	D_2/3_: 74.1 ± 6.7 (c), 70.1 ± 6.3 (p), 57.6 ± 6.7 (t), 51.3 ± 9.2 (fc), 58.4 ± 3.0 (tc)	9.9 (s), 12.2 (p), 18.9 (t), 24.3 (fc), 18.2 (tc)	89.1 (s), 109.8 (p)	Concentration reported for raclopride scans; lower in FLB457. No preferential extrastriatal binding of ARI
8	(22)	[^11^C]raclopride and [^18^F]FDG	RCT, PET and fMRI study with single dose of aripiprazole after fasting, sampling before scans	*N* = 15; healthy volunteers; age 23.1 ± 2.4; 100% males	12.4 ± 11.4 (2–30)	15.0 ± 14.3	D_2/3_: 50.2 ± 22.0 (s)	NA	NA	Reaction times in working memory task and metabolic change in frontal lobe pos. corr. with D_2_ occup.
9	(25)	[^11^C]raclopride	Cohort study; PET and fMRI scans performed after flexible ARI; trough samples in the steady-state	*N* = 7; SCZ (DSM-4); age 32; 28.6% males	14.2 ± 12 (2–30)	289.9 ± 325.2	D_2/3_: 65.0 ± 8.6 (s)	NA	NA	Error rates and reaction time in working memory task pos. corr. with D_2_ occup.

*c, cortex; fc, frontal cortex; p, putamen; s, striatum; tc, temporal cortex; t, thalamus; NA, no information available; RCT, randomized-controlled trial; SCZ, Schizophrenia; SD, schizoaffective disorder.*

Yokoi et al. (9) published the first PET occupancy study with aripiprazole in 15 healthy volunteers, who were treated with fixed aripiprazole doses for a duration of 14 days. They documented a dose-dependent increase of D_2/3_ dopamine receptor occupancy, with a mean occupancy of 30% (caudate) and 34% (putamen) at a dose as low as 0.5 mg, that increased to 49 and 57% at 1 mg, 74 and 72% at 2 mg, 86 and 85% at 10 mg, and 92 and 86% at 30 mg. These authors measured plasma levels, but they did not calculate EC_50_ values. However, the plasma concentration/occupancy curve reported by Yokoi et al. (9) is very similar to the one published by Gründer et al. (18), indicating that the flat part of the curve begins at around 100 ng/ml.

Mamo et al. (23) quantified aripiprazole binding to three different receptor types in 12 patients with schizophrenia, who were treated with aripiprazole doses between 10 and 30 mg daily: D_2/3_ dopamine (with [^11^C]raclopride), 5-HT_2_ serotonin (with [^18^F]setoperone), and 5-HT_1A_ (with [^11^C]WAY100635). Even the lowest dose was associated with 85% D_2/3_ dopamine receptor occupancy, and the higher doses led to occupancies above 90%. Extrapyramidal side-effects were documented in two patients (with occupancy > 90%) in whom plasma levels were far above the mean for their dose (442 ng/ml and 663 ng/ml, respectively). 5-HT_2_ serotonin occupancy was in the medium range (54–60%), while 5-HT_1A_ receptors were occupied by less than 20% (23). The authors measured aripiprazole and dehydroaripiprazole plasma levels, but EC_50_ values were not reported. However, at the (lowest) 10 mg dose the mean aripiprazole level was 126 ng/ml (dehydroaripiprazole 35 ng/ml); later PET studies [(18, 26), see below] have consistently shown that at these plasma levels D_2/3_ dopamine receptor occupancy is close to 90%. Mizrahi et al. (24) described the same patient sample that Mamo et al. (23) have been investigating. These patients with schizophrenia were switched from olanzapine or risperidone to aripiprazole and both D_2/3_ receptor occupancy and subjective well-being (with the Subjective Wellbeing under Neuroleptics Scale, SWN) were measured. Although receptor occupancy was very high under aripiprazole treatment (82–99%), the SWN score increased significantly after switch from an antagonist to the partial agonist antipsychotic. Plasma levels were not reported (24).

D_2/3_ dopamine receptor occupancy was measured in 16 patients with schizophrenia or schizoaffective disorder on steady-state treatment with aripiprazole at doses ranging from 5 to 30 mg daily by Gründer et al. (18). D_2/3_ receptor occupancy was high already at 5 mg/day, and receptors were almost completely occupied above plasma levels of 100–150 ng/ml (Figure 2). EC_50_ values for the various brain regions examined ranged from 4 to 10 ng/ml, with 10 ng/ml for the putamen and 9 ng/ml for the caudate. This study is also the only one that reports EC_50_ estimates that are based on active moiety (aripiprazole + dehydroaripiprazole) concentrations of the drug (putamen 20 ng/ml, caudate 18 ng/ml). Aripiprazole’s main (active) metabolite, dehydroaripiprazole, also occupies the D_2/3_ receptor. Thus, a not negligible fraction of total occupancy (usually 20–30%) is attributable to dehydroaripiprazole binding. When one calculates EC_90_ values based on an EC_50_ value of 10 ng/ml for aripiprazole alone and 20 ng/ml for the active moiety, these values are 90 and 180 ng/ml, respectively (18).

Kegeles et al. (20) measured D_2/3_ dopamine receptor occupancy in 19 patients with schizophrenia or schizoaffective disorder, who were subchronically (minimum of steady dose: 10 days) treated with aripiprazole doses between 2 and 40 mg daily. Occupancy values were very high, ranging from a mean of 72% at 2 mg/day to 97% at 40 mg/day. Changes in the PANSS positive symptom subscale correlated positively with receptor occupancy in the striatum, but not in extrastriatal brain regions. Unfortunately, since plasma levels were not measured in two patients, these authors related occupancy values to doses rather than plasma levels. Thus, EC_50_ values are not reported. Instead, they calculated ED_80_ values (effective dose 80: the dose, that is associated with 80% occupancy). The mean ED_80_ from striatal regions was 5.6 mg and the mean ED_80_ from extrastriatal regions 3.9 mg. While this significant difference indicates a high binding in extrastriatal brain regions, the 1.7 mg difference is clinically meaningless. The study is in line with the one by Gründer et al. (18) insofar as it indicates that D_2/3_ receptors are almost completely occupied by aripiprazole at doses as low as 10 mg/day (20).

Takahata et al. (26) assessed striatal D_2/3_ receptor occupancy with [^11^C]raclopride and extrastriatal occupancy with [^11^C]FLB457. They administered single oral doses of 6 mg aripiprazole to 11 healthy male volunteers 150 min prior to the PET scan. While they could not find differential binding in striatal and extrastriatal regions, D_2/3_ occupancy was 74% in the caudate and 70% in the putamen. The corresponding mean plasma concentrations were 29.4 ng/ml for aripiprazole and 1.4 ng/ml for dehydroaripiprazole. Based on these values, the calculated EC_50_ values were 9.9 ng/ml for the striatum and 12.2 ng/ml for the putamen. However, Takahata et al. (26) based the calculation of their EC_50_ values on plasma concentrations of the parent (aripiprazole) compound only (K. Takahata, personal communication). Because the concentrations of the metabolite were so low in that study (the PET scan was started 150 min after administration of the drug), its contribution to total occupancy was most likely very small. With prolonged treatment, the effect of dehydroaripiprazole on EC_50_ estimates is substantial (18).

Ito et al. (19) administered single oral aripiprazole doses in the range between 3 and 9 mg to twelve healthy men. They measured D_2/3_ receptor occupancy with [^11^C]raclopride PET and dopamine synthesis capacity with L-[β-^11^C]DOPA. The mean striatal D_2/3_ occupancies were 55% (putamen) and 57% (caudate) at 3 mg, 69 and 73% at 6 mg, and 76 and 78% at 9 mg. Plasma concentrations of aripiprazole and dehydroaripiprazole were assessed separately. They were 12 + 0.4 ng/ml at 3 mg, 29 + 0.9 ng/ml at 6 mg, and 40 + 1.4 ng/ml at 9 mg. EC_50_ values are not reported by Ito et al. (19). However, from the reported data a value of approximately 10 ng/ml can be roughly estimated.

Kim et al. (22) assessed D_2/3_ receptor occupancy with [^11^C]raclopride PET in 15 healthy volunteers after administration of single oral aripiprazole doses. In addition, they measured glucose metabolism with [^18^F]FDG and assessed cognitive performance. Mean D_2/3_ receptor occupancy was 16% after 2 mg aripiprazole, 36% after 5 mg, 63% after 10 mg and 73% after 30 mg. The corresponding aripiprazole plasma concentrations (there is no information in the paper on determination of metabolites) were 2.6, 5.8, 13.2, and 35.4 ng/ml. Although these values were determined after single doses in healthy subjects, they are in line with the EC_50_ values of approximately 10 ng/ml determined after chronic treatment in patients with schizophrenia (18, 26). Greater striatal D_2/3_ receptor occupancy was associated with lower frontal glucose metabolism, and greater reduction in frontal metabolism corresponded to longer reaction times (22).

The same authors compared two different analytical approaches on data from 18 healthy subjects (21), who received the same single aripiprazole doses as those applied in Kim et al. (22). It has to be assumed that the subject samples in these two studies are overlapping. The mean D_2/3_ receptor occupancy in this somewhat larger sample was 30% after 2 mg aripiprazole, 54% after 5 mg, 72% after 10 mg and 82% after 30 mg. The authors calculated an EC_50_ of 11.1 ng/ml with the conventional pharmacodynamic model. When they applied a novel PK-PD model, they found a slightly lower EC_50_ of 8.6 ng/ml. This difference might be considered negligible for clinical purposes, and when taking into account that these values are omitting the contribution of the metabolite to total aripiprazole occupancy.

Shin et al. (25) measured D_2/3_ receptor occupancy in seven patients with schizophrenia and related striatal occupancy to cognitive performance. They found that patients with higher occupancy performed better in certain cognitive dimensions such as working memory and reaction time (25). While these authors determined aripiprazole plasma levels at times of the PET scans, they did not report EC_50_ values.

#### Conclusion for Clinical Practice

Among the three available partial dopamine agonist antipsychotics, by far the broadest molecular imaging database exists for aripiprazole. Nine PET studies have been conducted over the last 20 years. Although only two of them estimated EC_50_ values (18, 26), the evidence regarding a therapeutic reference range that can be derived from those studies is appealingly consistent. Above a threshold of approximately 100 ng/ml aripiprazole (parent compound only) D_2/3_ receptors are close to being completely occupied. When the active moiety (aripiprazole + dehydroaripiprazole) is considered, this value is 180 ng/ml.

The “Consensus Guidelines for Therapeutic Drug Monitoring in Neuropsychopharmacology: Update 2017” (17) reports a therapeutic reference range of 100 – 350 ng/ml for the parent compound and 150 – 500 ng/ml for the active moiety. The lower thresholds are in good agreement with the imaging-based values. The upper thresholds are somewhat arbitrary in nature, since much higher values are tolerated by many patients in clinical practice. However, there are hints in the literature that point to an increased EPS risk at higher plasma concentrations (20).

### Brexpiprazole

Two PET studies that measured D_2/3_ receptor occupancy are available for brexpiprazole (27, 28) (Table 2). One study was conducted in healthy subjects after the administration of single oral brexpiprazole doses (28), the second study assessed D_2_/D_3_ receptor occupancy as well as 5-HT_1A_, 5-HT_2A_ and serotonin transporter (SERT) occupancies in a total of 12 patients with schizophrenia after 10 days treatment (27).

**TABLE 2:** PET studies reporting D_2_ receptor occupancy and brexpiprazole (BXP) blood concentrations.

No	Author, year	PET Tracer	Design	Subjects	*Mean Dose (range) [mg/day]*	Mean BXP Conc. (range) [ng/ml]	Mean Receptor Occupancy (%)	EC_50_ [ng/ml]	EC_90_ (estimated from EC_50_) [ng/ml]	Comment
1	(28)	[^11^C]raclopride	Cohort study with dose response PET of BXP after single doses (phase 1). Plasma samples measured with HPLC	*N* = 15; healthy volunteers; age 33.9 ± 6.8; 93.3% males	2.68 (0.25–6)	32.5 ± 25.8	D_2/3_ (p and c): 0.25 mg: < 20; 2–4 mg: 59–75; 5–6 mg: 77–88	7.75 (c), 8.13 (p)	69.8 (c), 73.2 (p)	BXP AUC and c_*max*_ increased with dose, no ADR observed in study.
2	(36)	[^11^C]-(+)-PHNO, [^11^C]CUMI101, [^11^C]MDL100907, [^11^C]DASB	Cohort study comparing patients at baseline (unmedicated) and medicated, trough serum conc. at steady-state measured with HPLC	*N* = 12; SCZ (DSM-4); age 42 ± 8; 58.3% males	3.0 (1–4), at day 4–10	82 ± 59 (*N* = 7 from D_2_ diagram)	D_2/3_: 47.7 ± 38.5 SERT: −3 ± 15 5-HT_1A_: 4 ± 6 5-HT_2A_: 36.5 ± 20.9	22 (s)	198 (s)	Dose dependent binding for D_2_ and 5-HT_2A_ receptors, not detectable for D_3_. EC_50_ from non-linear model. Values for other models ranged up to 52 ng/ml.

*c, cortex; p, putamen; s, striatum; t, thalamus; SCZ, Schizophrenia.*

Wong et al. (28) administered single brexpiprazole doses in the range between 0.5 and 6 mg to 15 healthy subjects and determined D_2/3_ receptor occupancy with [^11^C]raclopride at two different time points post-dose (4 h and 23.5 h). The mean D_2/3_ receptor occupancy in putamen and caudate nucleus increased with increasing doses, with less than 20% at the 0.25 mg dose and values above 80% at the 6 mg dose. Receptor occupancy remained in the similar range 23.5 h after drug administration. At the clinically recommended brexpiprazole doses of 2–4 mg/day, D_2/3_ receptor occupancies ranged from 59 to 75% at 4 h and from 53 to 74% at 23.5 h post-dose. When the estimated attainable maximum occupancy E_*max*_ was unconstrained, it was 89% for the putamen and 95% for the caudate, with the corresponding EC_50_ values being 8.1 and 7.8 ng/ml, respectively (28). When E_*max*_ was constrained to 100%, EC_50_ was 11.5 and 9.0 ng/ml, respectively.

When the estimation of an EC_90_ value is conducted based on an EC_50_ of 10 ng/ml, EC_90_ is 90 ng/ml, with an EC_50_ of 9 ng/ml the estimated EC_90_ is 81 ng/ml, and with an EC_50_ of 11 ng/ml the estimated EC_90_ is 99 ng/ml. Thus, the study suggests that at brexpiprazole plasma concentrations of 80–100 ng/ml striatal D_2_/D_3_ receptors are almost completely occupied by the drug.

The second PET study with brexpiprazole was a multi-tracer study to characterize the compound’s binding to four different molecular targets: dopamine D_2_/D_3_, serotonin 5-HT_1A_ and 5-HT_2A_ receptors, and the serotonin transporter (SERT) (27). While D_2_/D_3_ receptor occupancy is usually measured with antagonist radiotracers like [^11^C]raclopride or [^18^F]fallypride, this study applied the agonist tracer [^11^C]-(+)-PHNO. [^11^C]-(+)-PHNO allows the differentiation of binding to D_2_ and D_3_ receptors, but it systematically underestimates D_2_ occupancy by about 20% compared to assessment with antagonist radiotracers (29). After 10 days of treatment of patients with schizophrenia with brexpiprazole, the mean D_2_ receptor occupancy was 64% following 1 mg/day and 80% following 4 mg/day. The corresponding estimated EC_50_ values were, depending on the brain region, between 22 and 52 ng/ml (27). From these numbers an EC_90_ value between 198 and 495 ng/ml can be derived. Thus, in this study, at the same plasma concentrations the measured D_2_ receptor occupancies are substantially lower than in the study published by Wong et al. (28). While brexpiprazole did not significantly occupy the 5-HT_1A_ receptor and the SERT, 5-HT_2A_ receptor occupancy was 28% following 1 mg and 45% following 4 mg brexpiprazole (27).

#### Conclusion for Clinical Practice

The two available molecular imaging studies are inconclusive with regard to their clinical implications. One study determined D_2_/D_3_ receptor occupancy after single brexpiprazole doses (28); the second study used an agonist radiotracer that systematically underestimates D_2_ receptor occupancy (27, 29). Taking this underestimation into account, it seems reasonable to believe that striatal D_2_/D_3_ receptors are almost or completely saturated at 80–100 ng/ml brexpiprazole in plasma, and probably even at lower concentrations. However, this has to be confirmed in a study in patients treated with multiple doses and with an antagonist radiotracer.

The “Consensus Guidelines for Therapeutic Drug Monitoring in Neuropsychopharmacology: Update 2017” (17) reports a therapeutic reference range of 40 – 140 ng/ml for brexpiprazole. Based on the available PET studies, the lower limit value would tend to be too low, while the upper limit value could also be exceeded in clinical practice.

### Cariprazine

Two PET studies quantified D_2_/D_3_ receptor occupancy under treatment with cariprazine, one in monkeys (30) and one in humans (13) (Table 3). Seneca et al. (30) studied the occupancy of D_2_ and D_3_ dopamine receptors and 5-HT_1A_ serotonin receptors after a single low and a single high cariprazine dose, respectively, in three monkeys. Girgis et al. (13) assessed the occupancy of D_2_/D_3_ receptors by cariprazine in eight patients with schizophrenia at various doses and time-points post-dose.

**TABLE 3:** PET studies reporting D_2_ receptor occupancy and cariprazine (CP) blood concentrations (*converted; conversion factor 2.34).

No	Author, year	PET tracer	Design	Subjects	*Mean Dose (range) [mg/day]*	Mean CP conc. (range) [ng/ml]	Mean receptor Occupancy (%)	EC_50_ [ng/mL] (*converted; conversion factor 2.34)	EC_90_ (estimated from EC_50_) [ng/ml] (*converted; conversion factor 2.34)	Comment
1	(30)	[^11^C]raclopride, [^11^C]MNPA, [^11^C]WAY-100635	Animal PET study after single doses of CP, plasma samples measured with HPLC	*N* = 3; healthy monkeys (macaca fascicularis) 3–4 kg weight	(a) 1–5 μg/kg (b) 30–300 μg/kg	(a) < 1.0 (b) 3.1–34.1	D_2/3_: 5–94% (antagonist); D_2/3_: 45–80% (agonist); 5-HT_1A_: 18–30%	NA	NA	Dose dependent occupancy of 5–90% of D_2_/D_3_ receptors in striatum of monkeys
2	(13)	[^11^C]-(+)-PHNO	Cohort study after single doses of CP, plasma samples measured with HPLC	*N* = 9; SCZ; age 42 ± 8; 58.3% males	4.5 (1–12), at day 5–15	12.4 ± 13.1	D_2_: 0.91; D_3_: 0.78; (regions accounted for: c, p, vs, t, globus pallidus, substantia nigra/ventral tegmental area)	D_2_: 4.14 ± 0.91*; D_3_: 3.32 ± 0.87*	D_2_: 37.26*; D_3_: 29.88*	Near complete D_2_ and D_3_ occup. after 12 mg for 2 weeks. One patient withdrew due to emesis. PK-PD analysis reports higher EC_50_ values of 9.0 (D_3_) and 30.5 (D_2_).

*c, cortex; p, putamen; t, thalamus; vs, ventral striatum; NA, no information available.*

Seneca et al. (30) in their study in three monkeys applied three different radiotracers: D_2_/D_3_ receptor occupancy was quantified both with an agonist ([^11^C]MNPA) and an antagonist tracer ([^11^C]raclopride), and [^11^C]WAY-100635 was used for assessment of 5-HT_1A_ receptor occupancy. A total of 15 PET examinations were carried out. Each monkey was subjected to a baseline examination and then scanned again after intravenous administration of either a low (1–5 μg/kg body weight) or a high (30–300 μg/kg) dose of cariprazine. Blood samples for determination of the plasma concentrations of cariprazine and its two main metabolites desmethyl- (DCAR) and didesmethyl cariprazine (DDCAR) were taken at prespecified time-points. At doses of 5 and 30 μg/kg cariprazine caused a dose-dependent D_2_/D_3_ receptor occupancy of approximately 45 and 80%, while the highest dose (300 μg/kg) was associated with 94% occupancy. Occupancy values did not differ for agonist and antagonist radiotracers. Occupancy of 5-HT_1A_ receptors was 10–20% at the lower doses, and it plateaued at 30% with the highest dose (30). Although the authors measured plasma levels of cariprazine and its metabolites, they did not calculate EC_50_ values. Therefore, an EC_90_ value cannot be calculated based on that study.

The second study assessed cariprazine’s occupancy of D_2_/D_3_ receptors in patients with schizophrenia (13). The radioligand used was the agonist tracer [^11^C]-(+)-PHNO, and the patients were scanned at baseline and on days 1, 4, and 15 of treatment with cariprazine between 1 and 12 mg/day. Plasma (and cerebrospinal fluid) samples were analyzed for concentrations of cariprazine, DCAR, and DDCAR. After treatment with the lowest cariprazine dose (1 mg/day), D_3_ occupancy was 76% (range 58–89%) and D_2_ occupancy 45% (range 14–64%). At the dose of 3 mg/day, the mean D_3_ and D_2_ receptor occupancies were 92% (range 86–96%) and 79% (range 68–88%), respectively. Thus, at those lower doses, cariprazine binding was more selective for D_3_ over D_2_ receptors. At higher doses, this selectivity is lost. The dose of 12 mg/day led to complete saturation of both receptor subtypes. Since both metabolites are pharmacologically active, estimation of EC_50_ values were carried out with active moiety values (cariprazine + DCAR + DDCAR). Also, EC_50_ estimation was conducted separately for D_2_ and D_3_ receptors and for acute (occupancy estimation on days 1 and 4) and for subchronic treatment (occupancy estimation on day 15).

After acute dosing, the EC_50_ was 0.61 ng/ml for the D_3_ and 0.76 ng/ml for the D_2_ receptor. After 15 days treatment, when more of the slow-forming active metabolites, especially DDCAR, have accumulated, the EC_50_ values were 1.64 ng/ml for the D_3_ and 5.56 ng/ml for the D_2_ receptor. This suggests greater D_3_ selectivity of cariprazine with longer treatment, which is most likely explained by the grater D_3_ selectivity of DDCAR. DDCAR, which has a very long half-life, develops very slowly during treatment. While cariprazine is the dominant compound during the first few days of treatment, the active moiety mainly consists of DDCAR and cariprazine during chronic treatment (13). From the EC_50_ values estimated at day 15, the corresponding EC_90_ values are 14.8 ng/ml for the D_3_ receptor and 50.0 ng/ml for the D_2_ receptor.

#### Conclusion for Clinical Practice

Only one human PET study that provides EC_50_ estimates has been published, and this was conducted with the agonist radiotracer [^11^C]-(+)-PHNO. PET studies with the antagonist radiotracers [^11^C]raclopride and [^18^F]fallypride have been published as abstracts only. While the available PET study in monkeys suggests that D_2/3_ receptor occupancy is similarly high when assessed with the agonist [^11^C]MNPA and the antagonist [^11^C]raclopride, the D_3_-preferring agonist [^11^C]-(+)-PHNO might still underestimate D_2_ occupancy (29). The study by Girgis et al. (13) suggests that D_3_ and D_2_ receptors are almost completely saturated at approximately 15 and 50 ng/ml. The “Consensus Guidelines for Therapeutic Drug Monitoring in Neuropsychopharmacology: Update 2017” (17) reports a therapeutic reference range of 10 – 20 ng/ml for cariprazine. However, the latter range is based on cariprazine levels only, while the EC_50_ values estimated by Girgis et al. (13) are based on active moiety values. A therapeutic reference range for the active moiety (cariprazine + DCAR + DDCAR) will be necessarily higher than one for the parent compound only (see discussion of aripiprazole above). However, due to a lack of data, such a reference range has not been defined yet.

## Discussion

Molecular imaging, especially with PET, has been used since the late 1980s for determination of rational antipsychotic dosing. These studies did not only demonstrate that the doses of some of the classical antipsychotics such as haloperidol over the first decades of their clinical use were irrationally high (31). They also showed that some of the newer (second-generation) antipsychotics were initially not dosed correctly. The best example is risperidone. This compound was approved and marketed for the treatment of schizophrenia in the United States in 1993 and soon thereafter throughout the world. The highest approved dose was 16 mg, and two-digit doses were quite commonly used during the first several years after market access (32). The first PET study with risperidone was published in the year of market entry (33). Three healthy volunteers were administered a single 1 mg oral dose of risperidone. The determined D_2/3_ receptor occupancy was approximately 50% even at this very low dose. Subsequent studies showed that the incidence of EPS rises at doses above 6 mg risperidone daily, the dose at which D_2/3_ occupancy crosses the 80% threshold in most patients (34). It took years for the results of these PET studies to change clinical practice of excessive doses, years in which many patients suffered unnecessary side effects due to incorrect dosages. Thus, since the mid-1990s at the latest, the characterization of target engagement of new antipsychotics has been part of their development program.

This is also true for the class of dopamine partial agonists. Aripiprazole was the prototype of this class of new drugs, it entered the market in 2002 in the United States. With the publication of the first PET study on this compound (9), it became immediately clear that the magnitude of its target engagement has to be interpreted differently from antagonist antipsychotics, and that it does not follow the “65 – 80% therapeutic window” rule for D_2_ antagonists (10) (Figure 1). Aripiprazole is still by far the most extensively studied partial agonist antipsychotic, and – as demonstrated in this paper – the data are very consistent in showing that more than 90% of all D_2/3_ dopamine receptors are occupied above a plasma concentration of approximately 100 ng/ml of the parent compound. Theoretically, substantially increasing the plasma concentration above this value is probably of no benefit to the patient. This is underlined by a recent dose-response meta-analysis that demonstrated that the 95% effective dose of aripiprazole is 11.5 mg/day and that its antipsychotic efficacy does not increase above this dose (35). The plasma concentration, however, can substantially vary at a given dose (18). Thus, monitoring of the plasma concentration is certainly a better tool for tailoring treatment to the individual patient. Although factors that characterize a patient individually, e.g., his psychopathology, are likely to influence the measurement of receptor availability, these influences are small and negligible compared to the effects of pharmacological treatment *per se*.

The situation is much less clear for the other two available dopamine partial agonist, brexpiprazole and cariprazine. As outlined in this paper, the few PET studies that have been published with these compounds, are somewhat inconclusive with regard to a therapeutic reference range. Specifically, a lower threshold at which almost complete occupancy of D_2/3_ receptors can be assumed, cannot be derived from these studies with sufficient certainty. It would be desirable if at least one PET study that met certain methodological standards were carried out when a new antipsychotic is launched on the market, or even before it is launched. A methodological standard procedure for PET studies aiming at supporting therapeutic concentration ranges has not been specified yet. Certainly, such investigations should be performed in a minimum number of patients (*n* = 15 or larger) who have been treated for a sufficient period of time (minimum steady-state) over the entire dose range. An antagonist should be used as the radiotracer ([^11^C]raclopride or [^18^F]fallypride), as extensive reference data are available for these. Studies with agonists as radioligands or those with preferential binding to D_3_ receptors could supplement the characterization in individual cases. Not only a large variance in reporting the results across studies, but also a considerable heterogeneity in the study populations (i.e., healthy volunteers vs. patients; dose and blood sampling designs; measurement of solely the major analyte vs. the analyte plus active metabolites) impede a comparability of the results. In terms of design, it has to be differentiated between studies that do or do not aim at linking PET findings with clinical effects. In order to be able to report a reliable relationship between receptor occupancy and clinical effects, the study designs have to be far more complex than most of the studies reviewed in this work (i.e., including a randomized, double-blind study phase).

In summary, this overview shows that molecular imaging is an excellent tool for characterizing antipsychotics in general and partial dopamine agonists in particular (Table 4). This is not just an academic exercise. Once the relationship between plasma concentrations of a substance and its binding to the molecular target in the brain has been clarified (which can be done with little effort), the determination of the plasma concentration in the individual patient allows for tailor-made treatment at the lowest possible cost.

**TABLE 4:** Main pharmacokinetic parameters derived from PET studies of aripiprazole, brexpiprazole and cariprazine.

Partial agonists and active metabolites	Recommendation to use TDM	Half-live (t_1/2_)	Therapeutic reference range	Laboratory alert level
**Aripiprazole**	Recommended	60–80 h	100–350 ng/mL	1,000 ng/mL
Aripiprazole plus dehydroaripiprazole		30–47 days	150–500 ng/mL	
**Brexpiprazole**	Useful	90 h	40–140 ng/mL	280 ng/mL
**Cariprazine**	Useful	50–120 h	10–20 ng/mL	40 ng/mL
N-desmethyl cariprazine				
N,N-didesmethyl cariprazine		2–3 weeks		

## Author Contributions

GG developed the first draft of the protocol. XH contributed to the writing of the manuscript, to the development of the search strategy, and critical appraisal. CS contributed with writing and critical appraisal. All authors have read and approved the final manuscript.

## Conflict of Interest

GG has served as a consultant for Allergan, Boehringer Ingelheim, Institute for Quality and Efficiency in Health Care (IQWiG), Janssen-Cilag, Lundbeck, Otsuka, Recordati, ROVI, Sage, and Takeda. He has served on the speakers’ bureau of Gedeon Richter, Janssen Cilag, Lundbeck, Otsuka, Recordati. He has received grant support from Boehringer Ingelheim, Lundbeck and Saladax. He is co-founder and/or shareholder of Mind and Brain Institute GmbH, Brainfoods GmbH, OVID Health Systems GmbH and MIND Foundation gGmbH. The remaining authors declare that the research was conducted in the absence of any commercial or financial relationships that could be construed as a potential conflict of interest.

## Publisher’s Note

All claims expressed in this article are solely those of the authors and do not necessarily represent those of their affiliated organizations, or those of the publisher, the editors and the reviewers. Any product that may be evaluated in this article, or claim that may be made by its manufacturer, is not guaranteed or endorsed by the publisher.
